# A mimicker of a halo nevus: A melanocytic nevus superimposed on idiopathic guttate hypomelanosis

**DOI:** 10.1016/j.jdcr.2025.03.005

**Published:** 2025-03-26

**Authors:** Jadesola Akanji, Jessica R. Williams, Cherie M. Young, Valerie D. Callender

**Affiliations:** a St. George’s University School of Medicine, True Blue, Grenada; b Callender Dermatology and Cosmetic Center, Glenn Dale, Maryland; c Department of Dermatology, Howard University College of Medicine, Washington, District of Columbia

**Keywords:** dermatoscopy, halo nevi, idiopathic guttate hypomelanosis, melanocytic nevus

## Introduction

Halo nevi (HN), also known as Sutton nevi or leukoderma acquisitum centrifugum, are benign melanocytic lesions occurring in approximately 1% of the population.^1^ They commonly appear in children and adolescents, with a mean onset age of 15 years, but incidence is atypical in adults over 40 years of age.^1^^,^^2^ A familial tendency for HN has been reported, and HN have been associated with autoimmune diseases such as vitiligo and Hashimoto thyroiditis.^1^ The uncommon appearance of HN is related to melanocyte destruction by CD4^+^ and CD8^+^ T lymphocytes in the lesion.^2^ HN classically present as 2 to 5 mm red to brown papules surrounded by a well-demarcated rim of depigmentation resembling a halo.^3^ However, fewer than 50% of HN display the classic presentation, making clinical diagnosis challenging.^3^ Given its rarity in older adults, new-onset HN after age 40 years should raise suspicion for melanoma and warrant a biopsy.^2^

Differentiating HN from other causes of leukoderma is essential. One common differential for guttate leukoderma in older populations is idiopathic guttate hypomelanosis (IGH), a benign finding in up to 80% of patients over 70 years of age, affecting both sexes, all races, and all skin phototypes.^2^

We report an unusual case of a melanocytic nevus superimposed on IGH, clinically mimicking the appearance of HN. The case underscores the importance of using dermatoscopy to clinically differentiate between melanocytic lesions that may otherwise appear indistinguishable on gross examination.

## Case report

**Fig 1:**
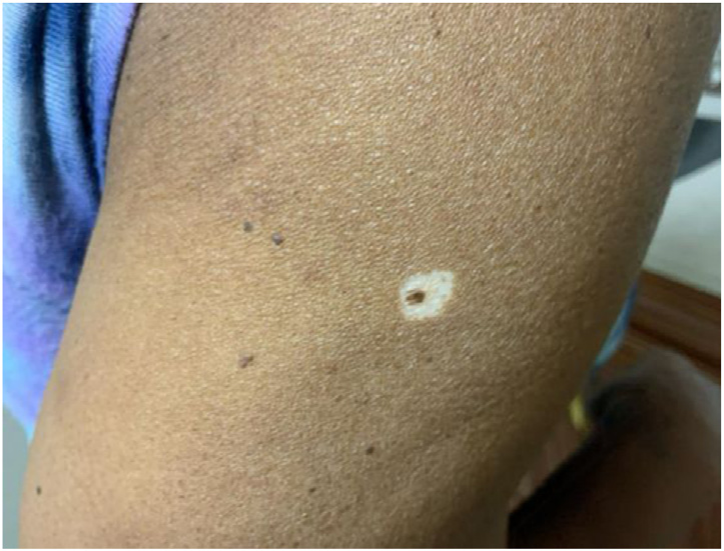
Melanocytic nevus superimposed on idiopathic guttate hypomelanosis on the right upper extremity of a 71-year-old woman at initial presentation.

A 71-year-old African American woman, Fitzpatrick skin type V, presented as a new patient. A full-body skin examination revealed bilateral upper extremity dyschromia with extensive hypopigmented macules, and hyperpigmented patches on the right side of the upper extremity. The hypopigmented macules were diagnosed as IGH, based on clinical examination. Additionally, a flesh-colored brown papule with apparent halo phenomenon on the posterior right side of the upper extremity was noted (Fig 1). The patient denied associated symptoms or recollection of the lesion’s onset.

**Fig 2:**
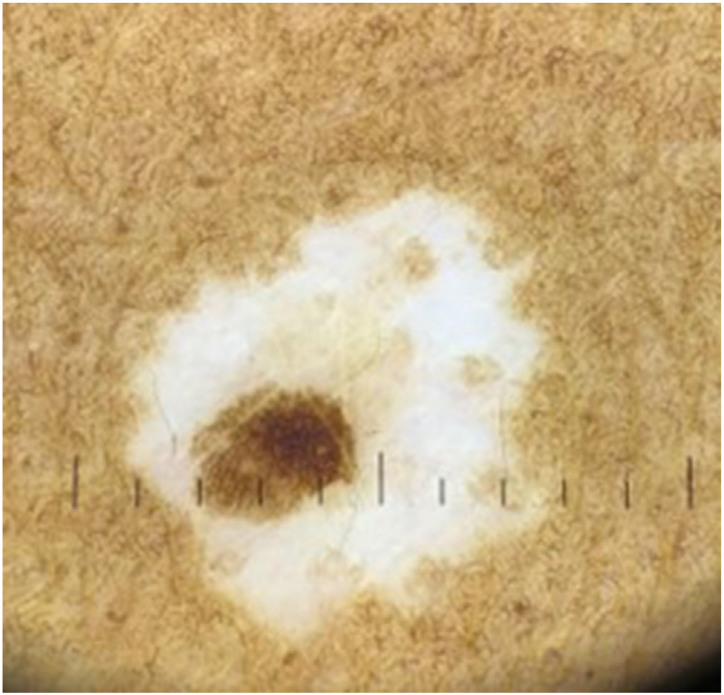
Dermatoscopic image showing a 2.5 mm nevus overlying 7 mm surrounding depigmentation. (Original magnification: ×10.)

Dermatoscopy revealed a 2.5 mm oval-shaped nevus composed of a delicate lace-like pattern with varying degrees of dispersed melanin (Fig 2). No concerning features such as dark globules or thickened strands were observed. Surrounding the nevus was a 7 mm irregular rim of porcelain-white macular depigmentation peripherally interspersed with faint skin-colored macules. Hair follicles were present in both the nevus and the depigmented region. No dermatoscopic features indicative of melanoma were identified within the lesions. Ultimately the patient was reassured and advised to monitor for any new changes in the lesion.

## Discussion

Melanocytic nevi (MN) are benign, well-circumscribed proliferations of melanocytes, presenting with a wide range of clinical and dermatoscopic features.^4^ Nevi can arise anywhere on the body; their appearance often varies depending on the anatomic site.

Our patient presented with a 2.5 mm flesh-colored brown nevus displaying a lace-like texture consistent with a reticular network pattern on dermatoscopy. Reticular patterned nevi typically feature a network of brown interconnected lines over a tan diffusely pigmented background. This pattern is commonly observed in junctional nevi or lentiginous nevi.^5^

Typical dermatoscopic features of HN include globular and/or homogenous patterns. In a study analyzing 138 HN images, 42% showed a combination of homogenous and globular patterns in different areas of the lesion, 23% displayed only the homogenous patterns, and 17% exhibited only the globular patterns.^1^ Reticular patterns were seen in only 9 out of the total 138 cases.^1^ This observation made the reticular nature of the patient’s lesion less consistent with that of HN and more consistent with that of MN.

HN result from chronic dermal inflammation, leading to melanocyte destruction via an active immune response.^3^ In its extreme form, the nevus is completely destroyed, leaving only a depigmented area,^3^ resembling vitiligo or regressing melanoma. Despite their similar pathogenesis, the risk of developing vitiligo in individuals with HN remains low.

Although benign, HN closely resemble regressing melanoma histologically, as both exhibit dense lymphocytic infiltrates, with the depigmentation progressively replacing nevus cells and melanoma showing infiltrates in areas where neoplastic cells have disappeared, mimicking a halo phenomenon.^6^ Given this overlap, distinguishing HN from regressing melanoma is crucial, and a skin biopsy is typically recommended in adults for definitive diagnosis.

IGH, a commonly acquired leukoderma, presents as multiple, discrete, sharply demarcated round or oval porcelain-white macules, measuring approximately 0.5 to 6.0 mm in diameter.^7^ IGH is often described as part of the natural aging process and may appear in both sun-exposed and sun-protected areas.^7^ These lesions frequently appear on sun-exposed areas, to include the extensor surfaces of the forearms and pretibial regions. IGH predominantly affects individuals over the age of 40 years, with an increased prevalence in older populations.^7^

IGH lesions exhibit various dermatoscopic patterns, including ameboid, feathery, petaloid, and nebuloid, with ameboid being the most common.^8^ The ameboid pattern features pseudopod-like projections around the macule’s periphery. Furthermore, a combination of dermatoscopic patterns can be seen in an IGH lesion. For example, a “cloudy sky” pattern, where smaller macules of varying shades of white coalesce into larger, often irregular, macules surrounded by a hyperpigmented network, has also been described.^8^

**Fig 3:**
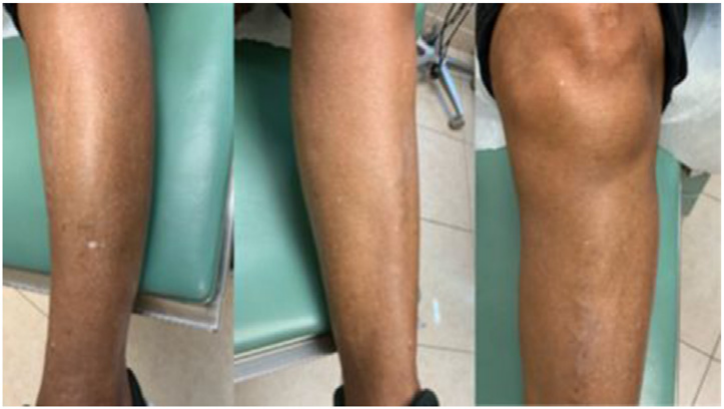
Multiple idiopathic guttate hypomelanosis noted on left lower extremity of patient on initial presentation.

Our patient’s lesion exhibited several features consistent with IGH. Numerous IGH lesions were noted during the full-body skin examination (Fig 3), making the presence of another IGH lesion in a sun-exposed area highly probable. The IGH lesion displayed an ameboid dermatoscopic pattern, marked by irregular peripheral extensions. In contrast, HN lesions are generally well-circumscribed and symmetric. Dermatoscopy further revealed distinct textural differences between the IGH lesion and the nevus, with the nevus demonstrating a lace-like reticular network pattern more characteristic of a benign melanocytic process.

In this case, dermatoscopy helped distinguish a nevus superimposed on IGH from a HN. Typical dermatoscopic features of HN include globular and/or homogenous patterns, commonly seen in benign MN. These features, along with reticular patterns, form the basis of dermatoscopic classification of MN. Identifying these dermatoscopic characteristics allowed us to differentiate the overlapping presentations of the nevus and IGH from a typical HN, avoiding the need for a biopsy. This case highlights the value of dermatoscopy in conjunction with an in-depth history and physical examination to reach an accurate diagnosis.

## Conflicts of interest

None disclosed.
